# Highly Accurate Visual Method of Mars Terrain Classification for Rovers Based on Novel Image Features

**DOI:** 10.3390/e24091304

**Published:** 2022-09-15

**Authors:** Fengtian Lv, Nan Li, Chuankai Liu, Haibo Gao, Liang Ding, Zongquan Deng, Guangjun Liu

**Affiliations:** 1 State Key Laboratory of Robotics and System, Harbin Institute of Technology, Harbin 150001, China; 2 State Key Laboratory of Robotics, Shenyang Institute of Automation, Chinese Academy of Sciences, Shenyang 110016, China; 3 Institutes for Robotics and Intelligent Manufacturing, Chinese Academy of Sciences, Shenyang 110169, China; 4 Key Laboratory of Marine Robotics, Shenyang 110169, China; 5 Beijing Aerospace Control Center Key Laboratory on the Technology of Space Flight Dynamics, Beijing 100190, China; 6 Department of Aerospace Engineering, Ryerson University, Toronto, ON M5B2K3, Canada

**Keywords:** Mars terrain, rovers, terrain classification, terrain visual features

## Abstract

It is important for Mars exploration rovers to achieve autonomous and safe mobility over rough terrain. Terrain classification can help rovers to select a safe terrain to traverse and avoid sinking and/or damaging the vehicle. Mars terrains are often classified using visual methods. However, the accuracy of terrain classification has been less than 90% in read operations. A high-accuracy vision-based method for Mars terrain classification is presented in this paper. By analyzing Mars terrain characteristics, novel image features, including multiscale gray gradient-grade features, multiscale edges strength-grade features, multiscale frequency-domain mean amplitude features, multiscale spectrum symmetry features, and multiscale spectrum amplitude-moment features, are proposed that are specifically targeted for terrain classification. Three classifiers, K-nearest neighbor (KNN), support vector machine (SVM), and random forests (RF), are adopted to classify the terrain using the proposed features. The Mars image dataset MSLNet that was collected by the Mars Science Laboratory (MSL, Curiosity) rover is used to conduct terrain classification experiments. The resolution of Mars images in the dataset is 256 × 256. Experimental results indicate that the RF classifies Mars terrain at the highest level of accuracy of 94.66%.

## 1. Introduction

**Figure 1:**
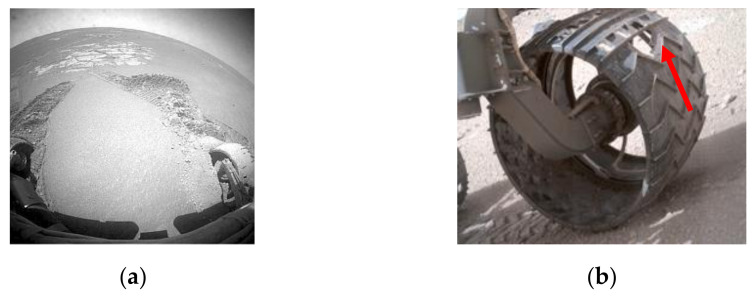
Risks to Mars rovers come from terrain: (**a**) Spirit rover sunk into the soil; (**b**) a puncture on a wheel of Curiosity.

Mars exploration motivates the search for extraterrestrial life, the development of space technologies, and the design of human missions and habitations [1]. Mars rovers are commonly used for Mars exploration. Mars rovers need to achieve mobility over rough challenging terrain for exploration missions. The greatest single source of risk for Mars rovers is Mars terrain. The Mars surface is covered with soft sand or hard gravel [2]. Although rovers can move fast on flat and nonslippery hard surfaces, they typically need to traverse a sandy/gravel surface at lower speeds. The soft sand may produce substantial wheel sinkage, causing the rover to become immobilized [3]. For example, the Spirit rover has sunk into the soil numerous times [4], as shown in Figure 1a. Hard gravel can produce significant vibration in a rover and damage the wheels. For example, the Curiosity rover has experienced an unexpectedly high damage rate on its wheel, as shown in Figure 1b. Due to the tribocorrosion caused by the interaction between the wheel and the terrain, this damage will gradually expand as the rover travels [5]. Such terrain hazards can only be identified visually. Knowledge of the terrain types will be useful for a rover to allow its path to be adjusted to avoid such hazards and improve its mobility. Thus, the detection of the terrain type is desirable for the control of high-mobility rovers.

Vision-based terrain classification is usually accomplished using pattern recognition. The images contain remote images and terrain images. Remote images are mainly used for crater identification and spatial-occurrence-based statistical analysis of various landforms. The terrain images are mainly used for the analysis of terrain traversability for rovers. Some areas of the terrain may be covered with dust. The dust is a soft terrain; it belongs to the sand terrain type. It can be recognized by terrain classification. According to the classification results, the areal occurrence of dust on Mars in terrain images can be calculated. The terrain images are treated as a signal source, and the color and texture of the images are extracted as features for training classifiers or classifying terrain. Numerous studies have focused on terrain classification based on vision. For example, references [6,7] extracted color features (sin(hue), cos(hue), saturation, and value), texture features (horizontal, diagonal, and vertical wavelet coefficients), and geometry features (average slope of the terrain, mean squared deviation of the points from the least-squares plane along its normal, variance in the height of the range data points, and the difference in height between the highest and lowest points within the patch) from binocular vision images. During the training phase, the features and corresponding terrain types were used to train three support vector machine (SVM) classifiers. During the classification phase, the features extracted from unknown-class terrain images were input into three SVMs to obtain three terrain classes. The three classification results were then fused using a naïve Bayes fusion approach to judge the terrain type [7].

A critical step to successfully building an image classifier is to extract and use informative features from the given images. For the difficulty of data acquisition for Mars terrain images, many studies tested terrain classification methods with roves’ fully operational duplicates in Earth conditions and then applied those methods to the actual rover. The image features that are often used for terrain classification in those studies include color features based on the RGB space [8,9,10,11,12], HSV [6,7,13,14,15], and Lab [16] spaces; Gabor features [12,17,18]; the contrast [10,11,12], correlation [12], energy [11,12,13], and consistency [12] of gray-level co-occurrence matrix (GLCM); SURF features [19,20]; Daisy features [19,20]; local binary patterns (LBP) [19,20,21]; local ternary patterns (LTP) [19,20,21]; local adaptive ternary patterns (LATP) [19,20]; contrast context histogram (CCH) [20]; and the mean [2,9,13,14,15,22], entropy [8,9,22], contrast [8,23], correlation [23], energy [8,23], homogeneity [8,9,23], and standard deviation [9,12,13,14,15,22] at the grayscale. These features can be used not only for terrain classification, but also for other image classification problems, such as animal classification. They usually have strong generality in the description of image textures, but lack specificity for terrain classification. The relevant research studies combine different general texture features and adopt feature selection technology to carry out terrain classification. However, the selected features may have been extracted without explicit prior knowledge of what properties represent the underlying scene that are reflected by the original image. It will reduce the classification accuracy for terrain classification. In these studies, the classifiers used include random forests (RFs) [2,12,18,19,20,21], SVMs [6,7,8,9,16,17,19,20], multilayer perceptron [13,14,15,19,20], LIBLINEAR [19,20], decision tree [19,20], naïve Bayes classifier [19,20], K-nearest neighbor (KNN) [13,14,15,17,19,20], extreme learning machine [17,24], batch-incremental regression tree model [22], probabilistic neural network [23], and multilayer feed forward neural network learning algorithm [10].

Several published studies have focused on Mars terrain classification [2,13,14,15]. Ono [2] used a set of NAVCAM images from MSL to study Mars terrain classification and extracted the following image features in gray, gradients, and range channels: the channel intensity and the intensity at an *x* and *y* offset from the pixel and the averages of the rectangles at random positions in the local context of the point of interest. RF is used to classify each pixel in the terrain image with an accuracy of the derivable terrain of 76.2%. Shang [13,14,15] investigated Mars terrain classification using a Plate South panorama image obtained from the panoramic camera on the Spirit rover. A fuzzy rough feature selection was applied to the color and gray statistics features to select essential informative features in representing Mars terrain images. In addition, SVM, KNN, and a decision tree were compared for classifying the terrain images, the results of which showed that the classification using an SVM with the selected features achieved an accuracy of 87.7%.

In most previous studies on Mars terrain classification, numerous traditional image features, which are often not specifically targeted for terrain classification issues when first proposed, also have been extracted without explicit prior knowledge of what properties represent the underlying scene that are reflected by the original image. This may cause the accuracy of the terrain classification to be insufficient at a rate of about 88%. To improve the accuracy of the terrain classification, novel image features specifically for the Mars terrain classification are proposed in this paper by analyzing the characteristics of different Mars terrains. Three classifiers, namely, KNN, SVM, and RF, are applied to obtain the terrain classification results. Experimental results show that the accuracy of the terrain classification adopting an RF classifier reaches 94.66% and that the recognition accuracy of each terrain type is higher than 92%.

The remainder of this paper is organized as follows. Section 2 introduces the types of Mars terrain images under investigation. In Section 3, the characteristics of different Mars terrains are analyzed and new image features for terrain classification are proposed. Section 4 provides a summary of the three types of learning classifier mechanism (namely, KNN, SVM, and RF) used to conduct the image classification. Section 5 discusses the experiment results of the Mars terrain classification. Finally, some concluding remarks are given in Section 6.

## 2. Mars Terrain Types

The Mars images used in this study are components of MSLNet [25]. The data set MSLNet consists of 6691 images that were collected by the Mars Science Laboratory (MSL, Curiosity) rover that was manufactured by NASA in USA. It contains wheel images, short-range terrain images, and long-range terrain images. The short-range terrain images refer to the terrain images close to the Curiosity rover, and the long-range terrain images refer to the terrain images far away from the Curiosity rover. We selected 100 short-range terrain images from this dataset to study Mars terrain classification. These images are all obtained under the natural lighting of Mars, without special lighting processing. The brightness of the pictures is not uniform, and some images have shadows.

**Figure 2:**
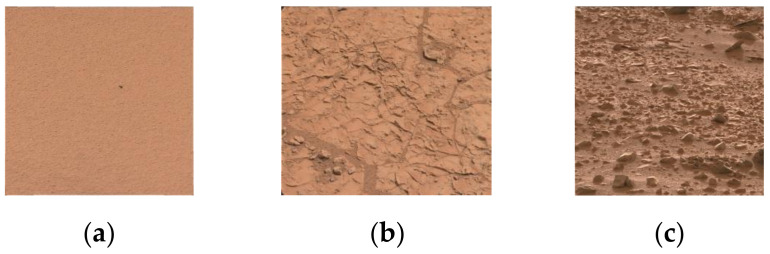
Mars terrain samples: (**a**) sandy terrain, (**b**) hard terrain, and (**c**) gravel terrain.

As shown in Figure 2, we identified the following three terrain types that need to be distinguished to operate a rover safely: sandy terrain (ST), hard terrain (HT), and gravel terrain (GT). ST is usually soft sand, HT is usually bedrock or slate that is difficult to deform, and GT is usually hard gravel. The three terrain types involve a majority of the terrain that the rovers encounter and need to be distinguished to operate a rover safely.

ST can easily cause significant sinkage because it is soft and compressible. The contact area between the wheel and terrain is large, and thus, the stress on the wheel is insufficient to cause an increase in the wheel cracking (Curiosity rover wheels) under a constant load. However, the Mars rovers may sink into the soil and become immobilized owing to a significant sinkage.

HT is hard and can create tiny deformations when the rovers move over it. The wheel can be regarded as having no sinkage. The HT can produce greater traction than a soft terrain, resulting in more effective mobility. Although rovers can move quickly on HT, the contact area between a wheel and terrain is extremely small, and therefore, the stress on the wheel is large under a constant load. This may induce stress concentration cracking at the chevrons of the grousers, resulting in crack growth.

GT is hard and uneven, and the rovers need to move slowly over it to decrease the vibrations produced and avoid damage to their components. In addition, the contact between the wheel and the sharp gravels can be treated as a point of contact. This can produce significant stress on the wheel rim, resulting in cracks. Round rock does not apply as high of a point load, but it can stress the grousers and cause cracks to propagate.

According to the above analysis, HT is the safest among the three types of terrains for a rover. Thus, it is the preferred one for the rover. The rovers need to adjust their control strategy and path according to the terrain types to improve their mobility and avoid damage. Therefore, the ability to classify these three terrain types will be a benefit for the rover motion.

## 3. Feature Extraction

As shown in Figure 2, The Mars terrain is usually red. Color features cannot be used to distinguish different terrain types clearly. To improve the accuracy of the terrain classification, we extract unique texture features to represent the underlying characteristics of a given image by analyzing the visual differences in the images for the different terrain types. These features consist of multiscale gray gradient-grade features (MSGGGFs), multiscale edges strength-grade features (MSESGFs), multiscale frequency-domain mean amplitude features (MSFDMAFs), multiscale spectrum symmetry features (MSSSFs), and multiscale spectrum amplitude-moment features (MSSAMFs).

### 3.1. Multiscale Gray Gradient-Grade Features

**Figure 3:**
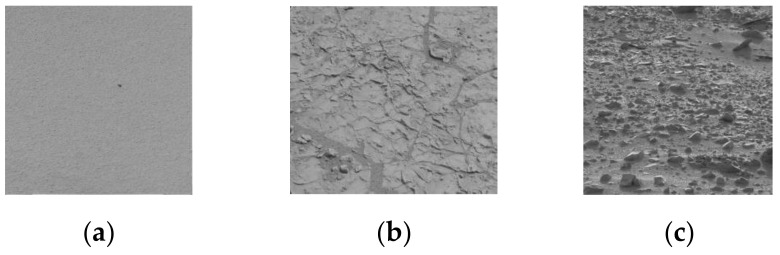
Mars terrain gray images: (**a**) sandy terrain, (**b**) hard terrain, and (**c**) gravel terrain.

It can be seen in Figure 3 that the changes in grayscale are the strongest for GT, followed by HT and ST. The more intense the changes are in the gray value, the larger the gray gradient. In this study, several thresholds were set to indicate the grayscale gradient levels. The pixel ratio of each gradient level in an image is extracted as the gray gradient-based features.

The gradient can represent a variation in the gray value of an image. The gradient expression of the pixel (*u*, *v*) is

(1) $$\left\{\begin{array}{l} g_{u}\left(u,v\right)=f\left(u+1,v\right)-f\left(u,v\right)\\ g_{v}\left(u,v\right)=f\left(u,v+1\right)-f\left(u,v\right) \end{array}\right.$$

(2) $$g\left(u,v\right)=\sqrt{g_{u}{}^{2}\left(u,v\right)+g_{v}{}^{2}\left(u,v\right)}$$

For any pixel point (*u*, *v*), a window with a scale of *n_i_* × *n_i_* centered on the pixel is selected to calculate the gradient value of each point in the window, obtaining a gradient image *g*. The number of pixels with gradient values greater than *th_gj_* is *N_gj_* in gradient image *g*. Here, *th_gj_* is the gradient threshold for indicating the grayscale gradient levels:

(3) $$th_{gj}=j\times d_{g}$$

where *j* indicates the gradient level, and *d_g_* represents the gradient value spacing of adjacent gradient levels.

Based on the gradient image, the pixel proportion $p_{gj}^{i}$ of the *j*-th gradient grade is extracted as the image feature.

(4) $$p_{gj}^{i}=N_{gj}/n_{i}{}^{2}$$

In this paper, *j* = 1, 2,…, 10, and *d_g_* = 5. Thus, the feature vector $P_{g}^{i}$ = [$p_{g1}^{i}$, $p_{g2}^{i}$, …, $p_{g10}^{i}$] is constructed with the window scale of *n_i_* × *n_i_*. Three windows of different scales are selected, and $P_{g}^{i}$ is extracted for each window. The multiscale gray gradient-based feature vector is $P_{g}^{}$ = [$P_{g}^{1}$, $P_{g}^{2}$, $P_{g}^{3}$].

### 3.2. Multiscale Edges Strength-Grade Features

**Figure 4:**
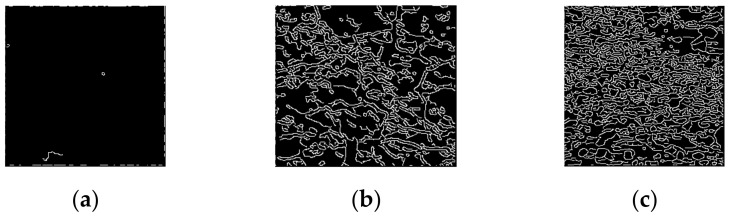
Extraction results of strong edges: (**a**) ST, (**b**) HT, and (**c**) GT.

The edges of the image can be extracted based on the gradient, for example, using the “Canny” algorithm. It is applied to extracting edges in the image in the present study. Figure 4 shows the results of edge extraction for the sample images. It can be seen in Figure 4 that the number of strong edges in the GT image is the largest, followed by the number of strong edges in an HT image and the smallest number of strong edges in an ST image.

For any pixel point (*u*, *v*), a window with a scale of *n_i_* × *n_i_* centered on the pixel is selected. The edges of the selected window are extracted using the “Canny” algorithm.

The edges at different edge strengths are extracted by changing the gradient threshold parameters of the “Canny” algorithm. The number of pixels of the edges extracted with the gradient threshold *th_ej_* is *N_ej_*:

(5) $$th_{ej}=j\times d_{e}$$

(6) $$p_{gj}^{i}=N_{gj}/n_{i}{}^{2}$$

where *j* indicates the edge strength level, and *d_e_* represents the gradient value spacing of the strength levels of the adjacent edge.

Based on the number of edges in the image, the pixel proportion $p_{ej}^{i}$ of the *j*-th edge strength grade is extracted as an image feature.

(7) $$p_{ej}^{i}=N_{ej}/n_{i}{}^{2}$$

In this study, *j* = 1, 2,…, 9, and *d_e_* = 0.1. Thus, the feature vector $P_{e}^{i}$ = [$p_{e1}^{i}$, $p_{e2}^{i}$, … $p_{e9}^{i}$] is constructed for a window with a scale of *n_i_* × *n_i_*. Three windows of different scales are selected, and the feature vector $P_{e}^{i}$ is extracted for each window. Therefore, we obtain a multiscale gray gradient-based feature vector, $P_{e}$ = [$P_{e}^{1}$, $P_{e}^{2}$, $P_{e}^{3}$].

### 3.3. Frequency Spectrum-Based Features

#### 3.3.1. Spectral Analysis for Images of Different Terrain Types

**Figure 5:**
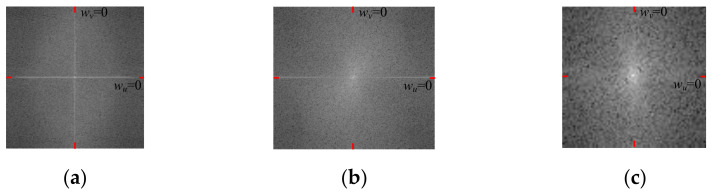
Spectrum images: (**a**) ST, (**b**) HT, and (**c**) GT.

Figure 5 shows the Fourier-transform spectrum images of an ST image, an HT image, and a GT image.

Owing to a uniform distribution of particles in the sand, the absorption and reflection of light in all directions of the terrain are approximately the same. The terrain image has a few strong boundaries and no obvious texture direction. For a spatial domain image of ST, the gray value distribution is concentrated, and the variation in the gray value and the gray gradient are both small. Therefore, the spectrum image of ST has the following characteristics: (1) a low brightness, (2) a nearly axisymmetric distribution along *w_u_* = 0 and *w_v_* = 0, and (3) an energy distribution concentrated in the low-frequency part. The bright spots in the spectrum image are concentrated in the low-frequency part. The low-frequency part has a high level of brightness, and thus, its amplitude is large. The high-frequency part has low brightness, and thus, its amplitude is small.

HT may have some pits or bulges on the surface owing to environmental factors, such as wind and light. Pits or bulges can form strong boundaries in an HT image. The gray value distribution of the spatial domain in a hard image is more concentrated than that in a GT image but is more scattered than that in an ST image. The gray value changes more gently than in a GT image but is more intense than that of an ST image. The gray gradient is smaller than that of a GT image but larger than that of an ST image. Therefore, the spectrum image of HT has the following characteristics: (1) more brightness than the spectrum image of an ST image, but more darkness than the spectrum image of a GT image, (2) no axisymmetry along *w_u_* = 0 or *w_v_* = 0, and (3) a slightly higher spectrum energy at low frequency than at high frequency. The bright spots in the spectrum image are more concentrated than those in the spectrum image of a GT image. The amplitudes of the high-frequency part are lower than those of the spectrum image of a GT image but higher than those of the spectrum image of an ST image.

Gravel on GT can obstruct light, forming a shadow on the terrain. An uneven distribution of gravel creates a large difference in the absorption and reflection of light for different directions of the terrain. The gray value distribution of the image is dispersed, the gray level changes drastically, and the gray gradient is large. Therefore, the spectrum image of ST has the following characteristics: (1) high brightness, (2) no axisymmetry along *w_u_* = 0 or *w_v_* = 0, and (3) a similar spectrum energy of the low-frequency and high-frequency parts. The bright spots in the spectrum image are scattered. The low- and high-frequency parts are both high in brightness and large in amplitude. The terrain will show obvious texture features in a single direction.

Three types of frequency spectrum-based features are extracted to represent the differences in frequency spectrum images of the three types of terrains.

#### 3.3.2. Multiscale Frequency-Domain Mean Amplitude Features

For any pixel point (*u*, *v*), a window with a scale of *n_i_* × *n_i_* centered on a pixel is transformed into the frequency domain to obtain a frequency spectrum image. The mean amplitude of the frequency spectrum image is

(8) $$p_{A}^{i}=\sum_{j=1}^{n_{i}}\sum_{k=1}^{n_{i}}A(u,v)/n_{i}{}^{2}$$

where *A*(*u*, *v*) is the amplitude of the point (*u*, *v*) in a frequency spectrum image.

Under windows of three different scales, a multiscale frequency-domain mean amplitude vector, $P_{A}$ = [$p_{A}^{1}$, $p_{A}^{2}$, $p_{A}^{3}$], of a terrain image is constructed.

#### 3.3.3. Multiscale Spectrum Symmetry Features

The terrain spectrum is divided into four parts, as shown in Figure 5. The spectrum symmetry along *w_u_* = 0 or *w_v_* = 0 in a *n_i_* × *n_i_* window is evaluated using the following features:

(9) $$\left\{\begin{array}{l} p_{Fx}^{i}=\left|m_{F}^{i1}-m_{F}^{i2}\right|\\ p_{Fy}^{i}=\left|m_{F}^{i1}-m_{F}^{i4}\right|\\ p_{\sigma x}^{i}=\left|\sigma_{F}^{i1}-\sigma_{F}^{i2}\right|\\ p_{Fy}^{i}=\left|\sigma_{F}^{i1}-\sigma_{F}^{i4}\right| \end{array}\right.$$

(10) $$\left\{\begin{array}{l} m_{F}^{i1}=\sum_{x=0}^{n}\sum_{y=0}^{n}\left|F\left(u,v\right)\right|/n_{i}^{2}\\ m_{F}^{i2}=\sum_{x=-n}^{0}\sum_{y=0}^{n}\left|F\left(u,v\right)\right|/n_{i}^{2}\\ m_{F}^{i4}=\sum_{x=0}^{n}\sum_{y=-n}^{0}\left|F\left(u,v\right)\right|/n_{i}^{2} \end{array}\right.$$

(11) $$\left\{\begin{array}{l} \sigma_{F}^{i1}=\sqrt{\sum_{x=0}^{n}\sum_{y=0}^{n}{\left(\left|F\left(u,v\right)\right|-m_{F}^{i1}\right)}^{2}}/n_{i}^{2}\\ \sigma_{F}^{i2}=\sqrt{\sum_{x=-n}^{0}\sum_{y=0}^{n}{\left(\left|F\left(u,v\right)\right|-m_{F}^{i2}\right)}^{2}}/n_{i}^{2}\\ \sigma_{F}^{i4}=\sqrt{\sum_{x=0}^{n}\sum_{y=-n}^{0}{\left(\left|F\left(u,c\right)\right|-m_{F}^{i4}\right)}^{2}}/n_{i}^{2} \end{array}\right.$$

where $m_{F}^{i}$ and $\sigma_{F}^{i}$ represent the mean value and standard deviation of the terrain spectrum at the scale of *n_i_* × *n_i_*.

For any pixel point (*u*, *v*), the spectrum symmetry features are extracted under the windows of three different scales. Thus, a multiscale spectrum symmetry feature vector, ***P***s = [$p_{Fx}^{1}$, $p_{Fx}^{2}$, $p_{Fx}^{3}$, $p_{Fy}^{1}$, $p_{Fy}^{2}$, $p_{Fy}^{3}$, $p_{\sigma x}^{1}$, $p_{\sigma x}^{2}$, $p_{\sigma x}^{3}$, $p_{\sigma y}^{1}$, $p_{\sigma y}^{2}$, $p_{\sigma y}^{3}$], is constructed.

#### 3.3.4. Multiscale Spectrum Amplitude-Moment Features

The amplitude moment for a pixel in the spectrum is defined as the result of the pixel amplitude multiplied by the distance between it and the center bright spot. The spectrum amplitude-moment feature in an *n_i_* × *n_i_* window is expressed as follows:

(12) $$p_{m}^{i}=\sum_{j=1}^{n_{i}}\sum_{k=1}^{n_{i}}A(u,v)\cdot d(u,v)/n_{i}{}^{2}$$

where *d*(*u*, *v*) is the distance between the pixel (*u*, *v*) and the center bright spot.

Three windows of different scales are selected. Thus, a multiscale spectrum amplitude-moment feature vector, $P_{m}$ = [$p_{m}^{1}$, $p_{m}^{2}$, $p_{m}^{3}$], is extracted for each pixel in the terrain image.

## 4. Terrain Classification Methods

A feature vector, ***P*** = {***P****_g_*, ***P****_e_*, ***P****_A_*, ***P****_s_*, ***P****_m_*} = {*p*_1,_ *p*_2,_…, *p_l_*}, with *l* components is obtained through feature extraction. Terrain classification is achieved by combining classifiers and the proposed feature vector ***P***.

### 4.1. K-Nearest Neighbor

The KNN algorithm was first proposed by Cover and Hart as a nonparametric classification algorithm [26] and has been widely used in various fields of pattern recognition and data mining. The idea of the KNN algorithm is as follows: given a sample ***P****_a_* to be classified, *K* neighbors of a given training sample set most similar to ***P****_a_* are first found. The types of these neighbors are weighted using the similarity between ***P****_a_* and each of its neighbors, where the similarity is typically measured based on the Euclidean distance metric (although any other distance metric may also work). The classification of the sample ***P****_a_* is then determined with the greatest number of votes among the *K*-nearest type labels. The similarity between ***P****_i_* and ***P****_j_* can be calculated using the Euclidean distance, as shown in the following equation:

(13) $$d(P_{i},P_{j})=\sqrt{\sum_{x=1}^{l}{\left(p_{ix}-p_{jx}\right)}^{2}}$$

where *l* denotes the length of the feature vectors ***P****_i_* and ***P****_j_*. When classifying the sample ***P****_a_* ={*p_a_*_1_, *p_a_*_2_…, *p_al_*}, first, calculate the distance *d*(***P****_a_*, ***P****_i_*) between the sample ***P****_a_* and each sample of the training set, and then find the samples ***P***_min1_, ..., ***P***_min*K*_ with the smallest *K d*(***P****_a_*, ***P****_i_*), where the corresponding category is *c*(***P***_min1_) …, *c*(***P***_min*K*_), $c\left(P_{\min j}\right)\in c$. Ultimately, the type of ***P****_a_* is calculated using the following equations:

(14) $$c_{knn}\left(P_{a}\right)=\underset{c_{i}\in c}{\arg\max}\sum_{j=1}^{K}\delta \left(c_{i},c\left(P_{\min j}\right)\right)$$

(15) $$\delta \left(c_{i},c\left(P_{\min j}\right)\right)=\left\{\begin{array}{ll} 1 & c_{i}=c\left(P_{\min j}\right)\\ 0 & c_{i}=c\left(P_{\min j}\right) \end{array}\right.$$

The advantage of the KNN classification algorithm is its simplicity and easy implementation, as well as its strong robustness and high accuracy. However, the number of calculations during the classification process is large. The selection value of the parameter *K* has a significant influence on the classification result. If *K* is too large, it may cause too many samples of other types for a nonclassified sample among *K* samples, which results in an incorrect classification prediction. If *K* is too small, the number of neighbors of a nonclassified sample is small. The classification is seriously affected by noise, reducing the classification accuracy.

### 4.2. Support Vector Machine

**Figure 6:**
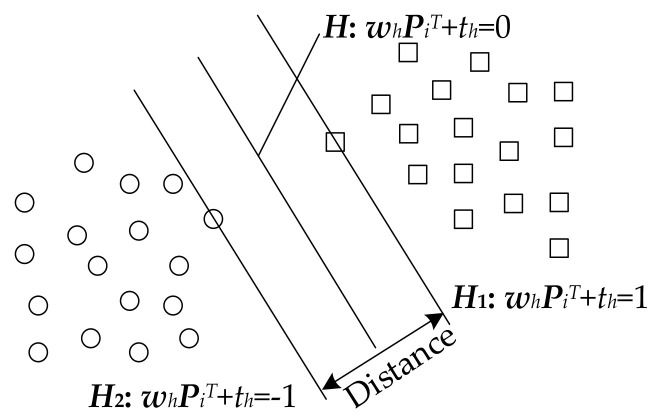
Schematic optimal separating hyperplane.

SVM dichotomizes data based on statistical learning theory [26]. The idea is to construct an optimal separating hyperplane in the feature space so that the plane can separate the two types of data, and the interval between the two types is the largest, as shown in Figure 6.

The training set is {***P****_i_*, *c_i_*}, $P_{i}\in R^{n},c_{i}\in \left\{\pm 1\right\}$. The equation of the hyperplane ***H*** is ***w***_h_***P****_i_^T^* + *t_h_* = 0. The plane ***H***_1_ is parallel to ***H*** and passes through the point closest to ***H*** in the first type. The plane ***H***_2_ is parallel to ***H*** and passes through the point closest to ***H*** in the second type. To eliminate the influence of singularities on hyperplane generalization, the slack variable *ξ_i_* is introduced. The construction of the optimal hyperplane can be transformed into the following convex quadratic programming problem:

(16) $$\begin{array}{l} \min\left(\frac{1}{2}{\left\|w_{h}\right\|}^{2}+\sum_{i=1}^{n}C_{s}\xi_{i}\right)\\ s.t.c_{i}\left(w_{h}P_{i}+t_{h}\right)-1\geq 0\;i=1,2,\ldots ,n_{s} \end{array}$$

where *n_s_* is the number of samples in the training set. *C_s_* is the penalty factor. Using a Lagrange multiplier, Equation (16) can be converted to the following dual problem:

(17) $$\begin{array}{l} W_{S}\left(\alpha \right)=\sum_{i=1}^{n}\alpha_{Li}-\frac{1}{2}\sum_{i=1}^{n}\alpha_{Li}\alpha_{Lj}c_{i}c_{j}\left(P_{i}P_{j}\right)\\ s.t.\sum_{i=1}^{n}\alpha_{Li}c_{i}=0\;\;\;0\leq \alpha_{Li}\leq C_{sL},\;\;\;i=1,2,\ldots ,n_{s} \end{array}$$

where *α_Li_* is the Lagrange multiplier. The *α_Li_* is not equal to zero for all points in ***H***_1_ and ***H***_2_. Therefore,

(18) $$w_{h}=\sum_{i=1}^{n}\alpha_{Li}c_{i}P_{i}$$

(19) $$t_{h}=c_{j}-\sum_{i=1}^{n}c_{i}\alpha_{Li}\left(P_{j}\cdot P_{i}\right)$$

The trained SVM is

(20) $$c_{SVM}\left(P_{a}\right)=\mathrm{sgn}\left(\sum_{i=1}^{n}c_{i}\alpha_{Li}\left(P_{a}\cdot P_{i}\right)+t_{h}\right)$$

For linearly indivisible data, the kernel function is usually used to transform the feature space to make the data linearly separable in the new feature space. The corresponding classifier is

(21) $$c_{SVM}\left(P_{a}\right)=\mathrm{sgn}\left(\sum_{i=1}^{n}c_{i}\alpha_{Li}K_{svm}\left(P_{a}\cdot P_{i}\right)+t_{h}\right)$$

where $K_{svm}\left(P_{a}\cdot P_{i}\right)$ stands for kernel function. It contains a linear kernel function, polynomial kernel function, radial basis kernel function, and sigmoid kernel function.

For the classification of *s_c_* types (*s_c_* > 2), the following three methods are usually adopted:

(1) For any type *c_i_*, SVM is constructed to realize the separating hyperplane of type *c_i_* and other types. A total of *s_c_* classifiers need to be constructed.
(2) An SVM classifier is constructed for any two types. A total of *s_c_* (*s_c_* − 1)/2 SVM classifiers are constructed. The *s_c_* (*s_c_* − 1)/2 results are obtained when classifying a sample. The type of a sample is determined by voting.
(3) Modify the SVM objective function to satisfy the multivalue classification.

### 4.3. Random Forests

Random forests [26] are evolved by combining the bagging algorithm with the decision tree algorithm. The bagging extracts *w* subsamples from the original database through a sampling with playback and then trains *m* base learners with *w* subsamples to reduce the variance in the model. However, not only random forests randomly extract subsamples from the original dataset, they also randomly select *t* features instead of selecting the optimal feature from all features to segment the nodes when training each base learner, further reducing the number of nodes. The subset of *t* features is different for each node. The variance in the model is lower. The basic learner used in random forests is the CART decision tree.

The smaller the sample subset size *w* of the random forest selection is, the smaller the variance in the model is, but the larger the deviation is. Therefore, in a practical application, we usually adjust the parameters through cross validation to obtain an appropriate sample subset size. The classification steps of random forests are as follows:

(1) Random samples: randomly select *w* samples from a sample set using bootstrap.
(2) Random features: *t* features are randomly selected from all features, and the best partitioning features are selected as nodes to establish a CART decision tree.
(3) Repeat the above two steps *m* times; that is, build *m* CART decision trees.
(4) Then, *m* CART forms random forests. The classification result is decided through a vote using *m* classification results of CART. The type *c_rf_*(***P****_a_*) of an unclassified sample ***P****_a_* is
  (22) $$c_{rf}\left(P_{a}\right)=\underset{c_{i}\in c}{\arg\max}\sum_{j=1}^{m}\delta \left(c_{i},c_{j}\left(P_{a}\right)\right)$$
  (23) $$\delta \left(c_{i},c_{j}\left(P_{a}\right)\right)=\left\{\begin{array}{l} 1\;\;c_{i}=c_{j}\left(P_{a}\right)\\ 0\;\;c_{i}=c_{j}\left(P_{a}\right) \end{array}\right.$$
  where *c_j_*(***P****_a_*) denotes the output of the *j*-th CART decision tree. The characteristics of random forests are as follows: (1) the random forest model is prone to an overfitting in sample sets with serious noise. (2) The more partitioned features are prone to have a greater impact on the decision making of random forests, thus affecting the fitting model.

## 5. Experiment Verification

### 5.1. Comparison of Terrain Classification Results with Different Features

The window scales for feature extraction are selected as 5 × 5, 10 × 10, and 30 × 30. The test samples for the three terrain types are the same. The number of trees in the random forest is five.

**Figure 7:**
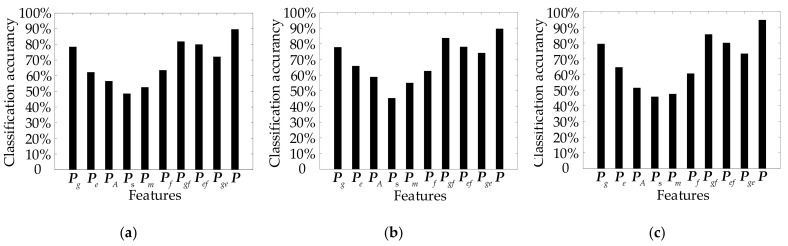
Terrain classification results of different groups of features: (**a**) classification results of KNN, (**b**) classification results of SVM, and (**c**) classification results of RF.

The classification accuracy of different groups of features is compared in this section. Half of the short-range images in MSLNet are treated as the training set to train terrain classifiers, and other short-range images are used to test the classification accuracy. Figure 7 shows the terrain classification results of different groups of features. The image features can be divided into five classes: MSGGGFs $P_{g}^{}$, MSESGFs $P_{e}$, MSFDMAFs $P_{A}$, MSSSFs $P_{s}$, and MSSAMFs $P_{m}$. Those features are used to classify terrain. Then, they are combined to conduct terrain classification. The combination features contain the frequency spectrum-based features (FBFs) $P_{f}$ (which are the combination of MSFDMAFs, MSSSFs, and MSSAMFs.), the combination of FBFs and MSGGGFs $P_{gf}$, the combination of FBFs and MSESGFs $P_{ef}$, the combination of MSGGGFs and MSESGFs $P_{ge}$, and the combination of all features ***P***. Table 1 shows the detail classification results using all features.

It can be seen in Table 1 that when single class features are used for terrain classification, the average classification accuracy of MSGGGFs is the highest, but lower than 80%. When the combination features of some classes are used for classification, the highest average classification accuracy is 85.48%, 9.18% lower than the average classification accuracy when all features are used. When all the features are used for terrain classification, the classification accuracy is the highest, and the average accuracy of terrain classification reaches 94.66%. In addition, the classification accuracy of HT is significantly improved. Therefore, every class feature plays an important role in terrain classification. Here, all features are combined to construct the feature vector for Mars terrain classification.

When all features are used for terrain classification, KNN, SVM, and RF classify HT at rates of 85.18%, 83.22%, and 92.27%, respectively; ST at rates of 92.65%, 91.56%, and 97.30%, respectively; and GT at rates of 90.64%, 93.96%, and 94.40%, respectively. Thus, the classification accuracies of the KNN, SVM and RF are approximately 89.63%, 89.58%, and 94.66%, respectively. The RF classifier has the highest classification accuracy and, thus, is the most beneficial for planetary rovers when adjusting their motion control strategy.

### 5.2. Comparison of Terrain Classification with the Use of Novel Features and Traditional Features

This section mainly compares the terrain classification results of the proposed image texture features and the traditional image texture features. The traditional image texture features used involve gray co-occurrence matrix texture features, Gabor texture features, spatial statistical texture features, LBP, and wavelet coefficients. Half of the short-range images in MSLNet are treated as the training set to train terrain classifiers, and other short-range images are used to test the classification accuracy. Table 2 shows the terrain classification results using the proposed image texture features and the traditional image texture features.

It can be seen in the results (Table 2) that for KNN, SVM, and RF classification methods, the classification accuracy using proposed image texture features is higher than that using traditional image texture features. The highest accuracy of the three methods is 85.44% when using traditional image texture features. Based on the proposed image texture features, the highest accuracy of terrain classification among the three methods is 94.66%. Therefore, the texture features proposed in this paper are helpful for improving the terrain classification accuracy.

### 5.3. Comparison of Terrain Classification Results with Different Classifiers

**Figure 8:**
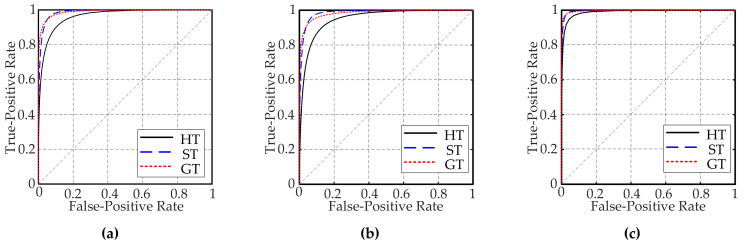
ROC curves: (**a**) ROC curve for the KNN classifier, (**b**) ROC curve for the SVM classifier, and (**c**) ROC curve for the RF classifier.

The classification method was tested using the short-range images in MSLNet. The classification methods were compared by cross validation of the resampling method in the statistical method. The data were divided into five parts, each containing three terrain types. Four parts were used as the training set, and the reserved one as the test set. The whole process was repeated five times in different ways. The classification results are shown in Table 3. The average value of the classification results five times cross validation was used to form the confusion matrix of the classification results to evaluate the performance of the classification methods. The detailed classification results of the KNN, SVM, and RF classifiers are shown in Table 4, Table 5 and Table 6. Two statistic indicators including precision PPV and specificity TNR were calculated and are shown in Table 7. Figure 8 shows receiver operating characteristic (ROC) curves for these three classifiers. Table 8 shows the area under the curve (AUC) values of all ROC curves.

The AUC values of the ROC curve for the KNN classification of GT, ST, and HT are 0.9915, 0.9892, and 0.9590, respectively. When identifying GT, ST, and HT using the SVM classifier, the AUC values are 0.9836, 0.9855, and, 0.9516, respectively. The AUC values for terrain classification achieved using the RF classifier are 0.9977, 0.9966, and 0.9859 for GT, ST, and HT, respectively. The ROC curves and AUC values show that KNN classifies GT with the best performance, although the true-positive rate (TPR) of ST is the highest, reaching 93.01%, as shown in Table 4. The precision of ST is 91.54%, which is less than the precision of GT at 94.22%. Similarly, the AUC values of the ROC curves show that both SVM and KNN also classify GT with the best level of performance. The classification accuracy of ST is higher than that of HT for all three classifiers.

If numerous STs and GTs (dangerous terrain) are misclassified as HT (safe terrain), the rovers will mistake a large amount of dangerous terrain as safe and, thus, choose such dangerous terrain to move over. This is not conducive to preventing the rover from sinking or incurring wheel damage. The misclassification rates of KNN, SVM, and RF are 15.23%, 17.62%, and 7.50%, respectively. Thus, RF misclassifies dangerous terrain as safe terrain at the lowest rate. This is more favorable for the safe driving of the rovers. ST is misclassified as HT by the KNN, SVM, and RF classifiers at rates of 6.65%, 7.26%, and 2.56%, respectively. Therefore, the RF classifier is the most beneficial for reducing the possibility of an ST being misclassified as an HT. This is most helpful for rovers sticking to avoid sinking owing to substantial wheel sinkage. However, the possibility of KNN or RF misclassifying ST as HT is greater, which is not conducive to avoiding a sinking accident. The proportions of GT misclassified as HT by KNN, SVM, and RF are 8.58%, 10.36%, and 4.94%, respectively. Therefore, RF is most beneficial for planetary rovers to avoid wheel damage caused by gravel. The rate of misclassification of HT as ST or GT by the RF classifier is 7.58%, which is lower than that for the KNN and SVM classifiers. Through the above analysis, the RF classifier achieves the best performance for Mars terrain classification. Thus, it is selected as a terrain classification classifier.

### 5.4. Classified Images

**Figure 9:**
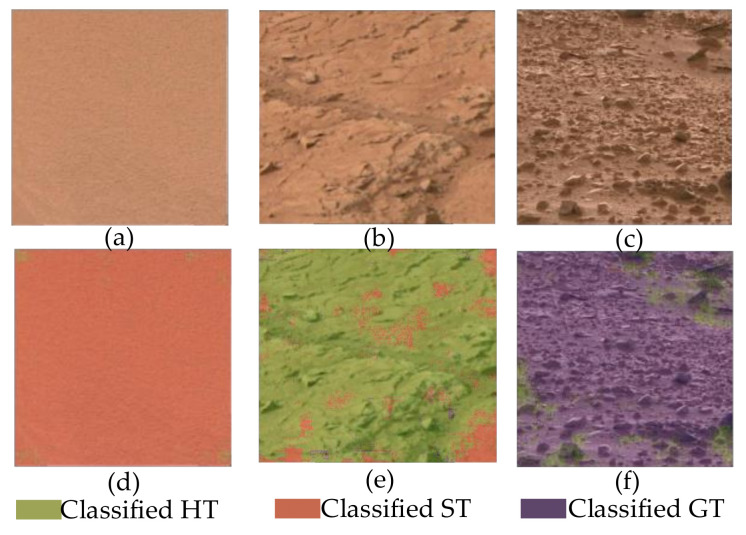
Classification of Mars single-type terrain images: (**a**) the original image of ST, (**b**) the original image of HT, (**c**) the original image of GT, (**d**) the classified image of ST, (**e**) the classified image of HT, and (**f**) the classified image of GT.

**Figure 10:**
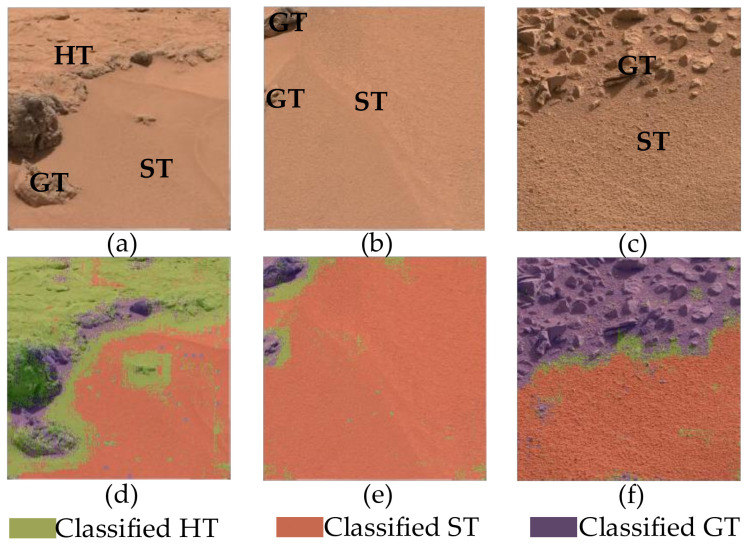
Classification of Mars mixture-type terrain images: (**a**) the original image 1, (**b**) the original image 2, (**c**) the original image 3, (**d**) the classified image 1, (**e**) the classified image 2, and (**f**) the classified image 3.

The ultimate task of this study is to classify Mars images and detect the terrain in such images. As an illustration, the images classified using the RF are shown in Figure 9 and Figure 10.

Most of the pixels in Figure 9d are classified as ST, and only a few pixels are classified as HT. Figure 9e shows that few pixels in the HT image are identified as ST, and other pixels are partitioned as HT. Almost all pixels in Figure 9f are recognized as GT; a small number of pixels are plotted as HT. The terrain classification is therefore quite efficient and accurate.

Figure 10a contains HT, GT, and ST. Figure 10b is the mixture of ST and GT. The components of Figure 10c are ST and GT. Figure 10d–f is the classified results. It can be seen that the method proposed in this study can better distinguish the terrain type of a region in an image with mixed-terrain types. In each terrain-type region, only a few pixels are classified incorrectly. The terrain classification has high accuracy. However, the pixels near the regional junction of different terrain types are continuously misclassified. Its features are coupled with the adjacent terrain types so that the differences between the extracted features and the features of two adjacent terrain types are large, resulting in misclassification. The width of the misclassification area is related to the feature scale.

### 5.5. Comparison with Other Classification Methods

**Figure 11:**
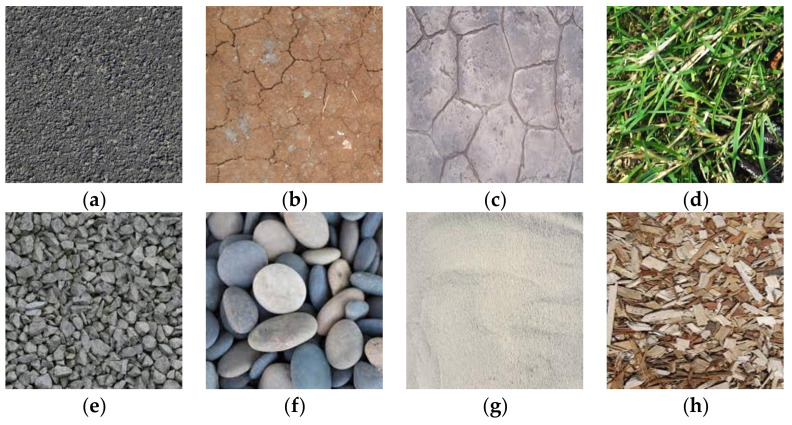
Sample images in Terrain8: (**a**) asphalt, (**b**) dirt, (**c**) floor, (**d**) grass, (**e**) gravel, (**f**) roc, (**g**) sand, and (**h**) wood chips.

The terrain image dataset Terrain8 [27] was used to evaluate the effectiveness of our method for visual terrain classification. Those images were all earth terrain images. Terrain8 consists of eight types of terrain: asphalt, dirt, grass, floor, gravel, rock, sand, and wood chips, as shown in Figure 11.

Based on the above experiment results, the RF classifier was selected as terrain classifier. The proposed method was compared with deep filter banks (DFBs) [27], hierarchical coding vectors (HCVs) [28], Fisher vector (FV) [29], LBP. Table 9 shows the classification results for five classification methods. Table 10 shows the classification accuracy of each type terrain for the proposed method in this paper.

It can been seen from Table 9 that the proposed method classified eight terrains with the highest accuracy, reaching 92.0%. In addition, the recognition rate of each type of terrain is not less than 85.9% by using the proposed classification method. Thus, the proposed method is also suitable for earth terrain classification.

### 5.6. Computational Requirements

The computational times of the terrain classification were obtained by using an image of 256 × 256 pixels. All algorithms in this work were implemented in the VS2015 version on an Intel Core i3-M380 2.53 GHz computer. Feature extraction requires 937.6 s per image. The training times of SVM and RF are 95.2 and 8.5 s, respectively. The times required for terrain classification by KNN, SVM, and RF are 892.9, 2.5, and 4.7 s respectively.

In the future, the method will be tested on board. The power consumption and running speed tests will be tested by using the experimental prototype of the Mars rover. The algorithm will be optimized and improved in consideration of the running speed, power consumption, and classification accuracy, so that it can be applied to the actual Mars rover, help the Mars rover to identify the terrain type, and select a safer driving path.

## 6. Conclusions and Discussions

In this study, a highly accurate method for in situ image-based Martian terrain classification is proposed. It is accomplished using newly proposed image features in conjunction with the RF classifier. The following conclusions were drawn:

(1) By analyzing the characteristics of the Martian terrain, novel image features, including multiscale gray gradient-based features, multiscale edges number-based features, multiscale frequency-domain mean amplitude features, multiscale spectrum symmetry features, and multiscale spectrum amplitude-moment features, specifically targeted for terrain classification issues are proposed. These features differ from traditional image features. Traditional image features can be used for image classification in numerous fields, and thus have universal applicability. However, they reducing the accuracy of some classification types, such as Martian terrain classification. The image features proposed in this paper are only for terrain classification, and they are beneficial for improving the accuracy of terrain classification, but they may not have universal applicability for image classification in other fields.
(2) The KNN, SVM, and RF classifiers were compared regarding terrain classification using the proposed image features. The experiment results show that RF classifies the Martian terrain with the highest level of accuracy, reaching 94.66%, and has a low proportion at 7.73% for dangerous terrain (ST and GT) classified as safe terrain (HT). It can therefore effectively help planetary rovers identify the terrain type and choose a safe terrain to traverse.

There are some issues need to be discussed.

(1) The Mars terrain types are divided into HT, ST, and GT, among which HT has better traversability, ST is easy to cause vehicle sinking, and GT is easy to cause hardware damage. The terrain classification method classifies the Mars terrain into HT, ST, and GT to help the rover identify the terrain type. Thus, the rover can select the terrain with better traversability to achieve safe driving. The terrain softness needs to be judged according to the wheel–terrain interaction force and the wheel sinkage. After obtaining the relevant data, a prediction system of terrain mechanical characteristics can be built by combining visual means. This is a future research direction. The system can be used to predict the terrain mechanical characteristics, including the softness and friction characteristics of the terrain, so as to facilitate the rover to judge the traversability of the terrain according to the terrain mechanical characteristics and select the best moving path.
(2) This paper is aimed at the study of the classification of the Mars terrain. The features proposed are based on the characteristics of the Mars terrain. The terrain classification method can be applied to any Mars rover. The dataset MSLNet collected by the Curiosity rover is used to test the terrain classification algorithm in this paper. The Mars terrain types are divided into HT, ST, and GT. Another image dataset of Mars terrain is similar to MSLNet. If this method is to be applied to another image classification, it needs to be analyzed according to specific problems. In this paper, the Earth surface image dataset Terrain8 is used to test the proposed classification method; the results show that this method is suitable for Earth terrain classification. However, it may not be suitable for the classification of animal images, since the feature extraction in this paper is aimed at terrain texture, which is different from animal texture. In the future, the following problems need to be studied.
(3) The Mars terrain classification method under complex lighting conditions will be studied and tested. The study on image enhancement will be carried out for different lighting conditions, including strong lighting, weak lighting, shadows formed by light occlusion, uneven lighting brightness, and so on, to increase the robustness and applicability of the algorithm.
(4) It can be seen from the experimental results that misclassifications often occur in the regional junction of different terrain types. To solve this problem, image segmentation technology can be introduced in the future to segment the different types of terrain regions and then conduct terrain classification to obtain the terrain type in every region to improve the classification accuracy of heterogeneous terrain junction regions.
(5) To realize the high-fidelity simulation and motion control of planetary rovers considering terramechanics, a terrain–environment perception system of a planetary rover can be built by combining vision-based terrain classification with the identification of the terrain properties. The system can help a planetary rover construct a knowledge base of the visual terrain features and terramechanics properties, and estimate the value domain of the terrain parameters using the terrain images where the rover will move.

## Figures and Tables

**Table 1:** Terrain classification results using all features ***P***.

Classifiers	Terrain Classification Accuracy			Mean Accuracy
	HT	ST	GT	
KNN	85.58%	92.65%	90.64%	89.63%
SVM	83.22%	91.56%	93.96%	89.58%
RF	92.27%	97.30%	94.40%	94.66%

**Table 2:** Comparison of terrain classification using different features.

Classification Methods	Features	Classification Accuracy
KNN	Proposed image texture features	89.63%
	Traditional image texture features	84.89%
SVM	Proposed image texture features	89.58%
	Traditional image texture features	83.27%
RF	Proposed image texture features	94.66%
	Traditional image texture features	85.44%

**Table 3:** Cross-validation results of terrain classification for three classifiers.

Classifiers	KNN	SVM	RF
1st test	90.02%	88.85%	95.18%
2nd test	90.27%	88.94%	95.29%
3rd test	90.28%	88.96%	95.27%
4th test	90.22%	88.84%	95.24%
5th test	90.18%	88.78%	95.20%
Average	90.19%	88.87%	95.24%
Standard error	0.0009	0.0007	0.0004

**Table 4:** Confusion matrix for Mars terrain classification results of the KNN classifier.

		Actual Terrain Types		
		HT	ST	GT
Classified terrain types	HT	86.46%	6.65%	8.58%
	ST	8.29%	93.01%	0.31%
	GT	5.25%	0.34%	91.11%

**Table 5:** Confusion matrix for Mars terrain classification results of the SVM classifier.

		Actual Terrain Types		
		HT	ST	GT
Classified terrain types	HT	84.78%	7.26%	10.36%
	ST	8.96%	92.46%	0.26%
	GT	6.26%	0.28%	89.38%

**Table 6:** Confusion matrix for Mars terrain classification results of the RF classifier.

		Actual Terrain Types		
		HT	ST	GT
Classified terrain types	HT	93.42%	2.56%	4.94%
	ST	4.92%	97.38%	0.15%
	GT	1.66%	0.06%	94.91%

**Table 7:** The secondary statistical indicators of the confusion matrix.

Classifiers	Terrain Types	Precision PPV	Specificity TNR
KNN	HT	85.02%	92.38%
	ST	91.54%	95.70%
	GT	94.22%	97.20%
SVM	HT	82.79%	91.19%
	ST	90.93%	95.39%
	GT	93.18%	96.73%
RF	HT	92.57%	96.25%
	ST	95.05%	97.46%
	GT	98.22%	99.14%

**Table 8:** Mars terrain classification AUC for the three classifiers.

		Terrain Types		
		HT	ST	GT
Classifiers	KNN	0.9590	0.9892	0.9915
	SVM	0.9516	0.9855	0.9836
	RF	0.9859	0.9966	0.9977

**Table 9:** Comparison of classification results of five classification methods.

Methods	Classification Accuracy
Proposed mothed	92.0%
DFB	89.8%
HCV	85.6%
FV	81.0%
LBP	78.3%

**Table 10:** Classification accuracy of each type of terrain for the proposed method.

Terrain Type	Classification Accuracy
Asphalt	93.6%
Dirt	94.1%
Grass	85.9%
Floor	93.9%
Gravel	94.0%
Rock	90.9%
Sand	91.6%
Wood chips	92.0%

## Data Availability

Not applicable.
